# Design and Implementation of a Wave Measurement System Based on Millimeter-Wave Radar Array

**DOI:** 10.3390/s26030859

**Published:** 2026-01-28

**Authors:** Zhijin Qiu, Yunfei Jiang, Bo Wang, Chen Fan, Yushang Wu, Zhiqian Li, Jing Zou, Bin Wang

**Affiliations:** 1 State Key Laboratory of Physical Oceanography, Institute of Oceanographic Instrumentation, Qilu University of Technology (Shandong Academy of Sciences), Qingdao 266001, China; qzj@qlu.edu.cn (Z.Q.); jiangyunfeiqlu@163.com (Y.J.); fanchenqlu@163.com (C.F.); little_wu@qlu.edu.cn (Y.W.); lizhiqian@qlu.edu.cn (Z.L.); zoujing@qlu.edu.cn (J.Z.); 2 School of Information Science and Technology, Qingdao University of Science and Technology, Qingdao 260011, China; 18661855732@163.com

**Keywords:** millimeter-wave radar array, radar ranging, ocean waves, zero-crossing method, spectral estimation method

## Abstract

Ocean waves are created by energy passing through water, causing it to move in a circular motion and have a crucial impact on the safety of ship navigation, offshore engineering construction, and marine disaster early warning. Therefore, developing high-precision, real-time wave observation technology to accurately obtain wave parameters is very important. This study employs a One-Vertical-Two-Inclined Millimeter-Wave Radar Array (1V2I-MMWRA) to observe wave parameters in the South China Sea. Based on the measured displacement time series, significant wave height, mean wave height, significant wave period, and mean wave period were estimated using both the zero-crossing method and spectral estimation. The system performance was validated against an air–sea interface flux buoy. Experimental results demonstrate that the zero-crossing method exhibits superior precision. The Root-Mean-Square Errors (RMSEs) for the aforementioned parameters were 0.13 m, 0.11 m, 0.81 s, and 0.46 s, respectively. In contrast, spectral estimation yielded higher RMSEs of 0.20 m, 0.16 m, 1.07 s, and 0.74 s, primarily attributed to increased deviations during typhoon passage. Furthermore, directional spectrum analysis reveals that peak frequency and Power Spectral Density (PSD) intensify with the strengthening of the typhoon, while estimated wave directions align closely with in situ measurements. These findings confirm the high reliability of the 1V2I-MMWRA under extreme conditions, highlighting its distinct advantages of lower power consumption and ease of deployment.

## 1. Introduction

Ocean waves constitute one of the most critical and complex oceanographic elements, significantly impacting marine forecasting, ocean engineering, disaster prevention and mitigation, the protection of maritime rights, and navigation safety. Consequently, the accurate measurement of waves is of paramount importance [1].

Currently, wave measurement techniques can be classified into contact and non-contact methods. Contact measurement is an observational approach in which instruments are directly deployed within the ocean wave field to obtain wave characteristic parameters. Common examples include bottom-mounted Acoustic Doppler Current Profilers and wave buoys. However, this approach faces challenges such as difficult deployment and limited measurement coverage [2,3]. Non-contact measurement involves positioning instruments at a distance from the wave field. Common methods include stereophotography, X-band radar, and satellite remote sensing. Stereophotography acquires wave data by continuously capturing images of the sea surface; these images are subsequently processed to calculate fundamental wave characteristic parameters. However, this method is characterized by high computational complexity and is limited by factors such as lighting conditions and camera resolution [4]. Traditional X-band wave-measuring radar systems are characterized by high system power consumption and exhibit a $180^{\circ }$ ambiguity in wave direction [5]. Satellite remote sensing offers the distinct advantage of extensive measurement coverage. However, regarding the measurement of detailed wave characteristics, this approach is constrained by low accuracy, compromised data reliability, susceptibility to adverse weather conditions, and high costs [6]. However, non-contact wave measurement methods are widely adopted as they significantly reduce the risk of equipment damage resulting from direct contact with seawater. This study leverages the inherent advantages of non-contact measurement while addressing the challenges of low accuracy, complex deployment, and high costs by proposing a novel approach for measuring wave characteristics using the 1V2I-MMWRA. The compact dimensions of millimeter-wave radar modules facilitate the design of a system that is lightweight and easy to deploy [7]. Operating within the frequency band of 60–64 GHz, corresponding to a wavelength of approximately 5 mm, the system achieves measurement accuracy at the millimeter level. The 1V2I-MMWRA facilitates the rapid and precise acquisition of wave surface variations while maintaining high immunity to meteorological interference. This operational robustness ensures reliable performance in all weather conditions, thereby significantly enhancing the fidelity of wave parameter retrieval [8,9,10].

Following the acquisition of distance information using the 1V2I-MMWRA, the data require subsequent processing to extract wave characteristic parameters. Currently, commonly used methods for calculating wave characteristic parameters, such as wave height and wave period, include the zero-crossing method and the spectral estimation method [11]. The zero-crossing method is widely employed due to its relatively simple data processing procedure and high accuracy. In a study by Kumar et al, the zero-crossing method was applied to the estimation of ocean wave characteristic parameters [12]. Furthermore, both the Waverider buoy developed by Datawell in the Netherlands and the TRIAXYS buoy from Canada employ the zero-crossing method to derive ocean wave characteristic parameters [13,14]. The spectral estimation method was initially applied in wave buoy observations. This approach relies on wave energy spectrum analysis and examines the energy distribution characteristics of ocean waves in the frequency domain to estimate key parameters, including wave height and wave period.

Regarding the estimation of wave directional spectra, numerous researchers have proposed diverse methods, including wave directional spectrum inversion algorithms based on instrument arrays and parametric estimation methods. Currently, the most common wave directional spectrum inversion algorithms based on instrument arrays include the Maximum Likelihood Method (MLM), the Extended Eigenvector Method (EEV), and the Bayesian Directional Spectrum Estimation Method (BDM). The MLM is the most commonly used method for wave directional spectrum estimation. This method is characterized by high computational efficiency, result stability, low sensitivity to noise, and satisfactory numerical accuracy. However, when the instrument array is situated in proximity to a reflecting surface, the directional resolution of the MLM may be compromised [15]. The EEV is capable of generating a wave directional spectrum in which the energy distribution is highly concentrated around the dominant wave direction; however, the range of applicability of the EEV still requires further verification [16]. The BDM can yield a smoother directional energy distribution and produce a more reliable and higher accuracy wave directional spectrum, provided that the number of array elements is no fewer than four, the quality of the cross-spectra is high, and the instrument deployment depth is not excessive. The main drawback of BDM is the long computational time [17]. The parametric estimation method is a simple and practical approach originally proposed by Longuet-Higgins. The parametric estimation method was subsequently refined by Borgman, Panicker, Borgman, and Hasselmann, who each proposed different truncated functions to represent the directional distribution, including truncated Fourier series, cosine power series functions, circular normal distributions, and normal distributions. Among these, the truncated Fourier series is the simplest [18,19,20,21].

This study presents a 1V2I-MMWRA-based wave measurement system designed to address the limitations of traditional equipment, such as cumbersome deployment, high power consumption, limited precision, and lack of all-weather capability. By acquiring high-frequency and high-precision range parameters measured in the one-vertical and two-inclined directions, wave characteristic parameters are estimated using a combination of the zero-crossing method, spectral estimation, and parameter estimation. Furthermore, sea trials were conducted to validate the feasibility of the measurement system and methods, providing a novel and efficient approach for wave measurement.

## 2. Materials and Methods

### 2.1. Data

**Figure 1:**
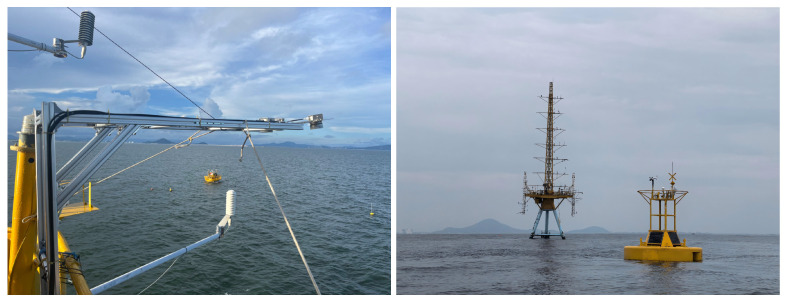
Deployment of the 1V2I-MMWRA and the air–sea flux buoy.

The data utilized in this study were acquired during a comprehensive offshore observation experiment conducted between 2 September and 30 September 2024. The experiment was performed at the offshore meteorological observation tower of the Bohe Marine Meteorological Field Experiment Base, CMA, located in the South China Sea ($111^{\circ }23^{\prime }3.314^{\prime \prime }$ E, $21^{\circ }26^{\prime }17.102^{\prime \prime }$ N). The observation tower is situated approximately 6.5 km offshore, where the surrounding water depth is about 15 m. Due to this considerable distance from the coastline, wave characteristics in the vicinity of the tower are minimally influenced by coastal effects. Furthermore, the fixed location and robust structure of the observation tower provide a stable platform for the deployment of the 1V2I-MMWRA. As shown in Figure 1, the 1V2I-MMWRA utilized in this study was installed on the platform level of the tower. This position offers an unobstructed field of view, facilitating continuous observation of the surrounding sea surface with high temporal resolution. To validate the accuracy and reliability of the wave characteristic parameters derived from the 1V2I-MMWRA, an air–sea flux buoy was deployed directly beneath the tower platform to serve as a synchronous reference instrument. The buoy is equipped with high-precision wave sensors capable of providing key wave characteristic parameters, including wave height, wave period, and wave direction. Through synchronous observations utilizing both the 1V2I-MMWRA and the air–sea flux buoy, a data acquisition framework for the cross-validation of wave parameters was established. This framework provided observational data for the subsequent analysis of radar retrieval accuracy and the investigation of data consistency under varying sea states.

**Figure 2:**
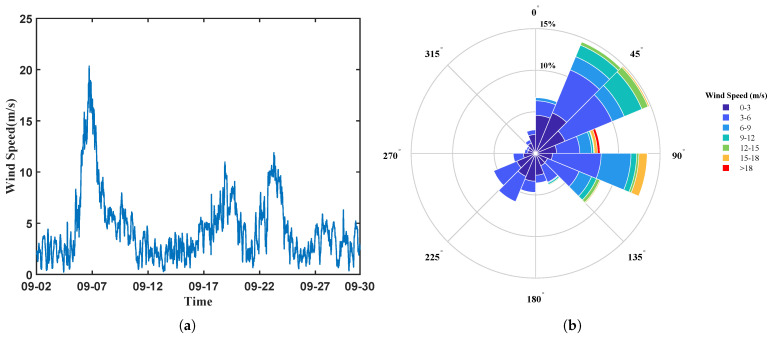
Statistics of Wind Variation During the Experiment: (**a**) Time series of wind speed in September; (**b**) Wind rose in September.

As shown in Figure 2, the wind conditions measured by the buoy in September are presented. Figure 2a displays the wind speed time series, from which it can be observed that Super Typhoon Yagi occurred around 7 September, and several high-wind events were recorded from 17 September to 27 September. Figure 2b depicts the wind rose, which visually summarizes the statistical distribution of wind speed and direction.

### 2.2. Core Module Design

**Figure 3:**
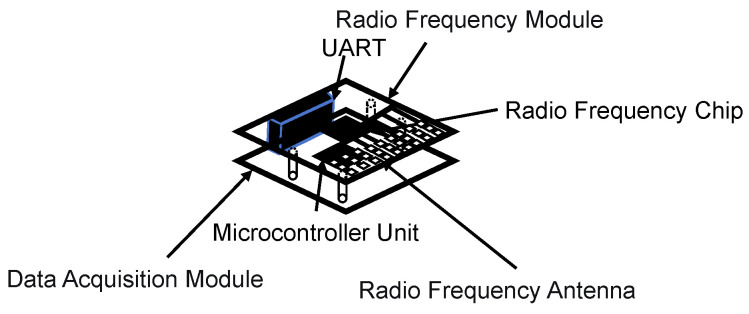
Schematic diagram of the core components of the millimeter-wave radar.

The millimeter-wave radar unit is the core module of the 1V2I-MMWRA. Each millimeter-wave radar unit comprises a Radio Frequency (RF) module and a data acquisition module. As shown in Figure 3, these modules are combined through stacking to become a single millimeter-wave radar core module. The key technical parameters of the radar are listed in Table 1.

**Figure 4:**
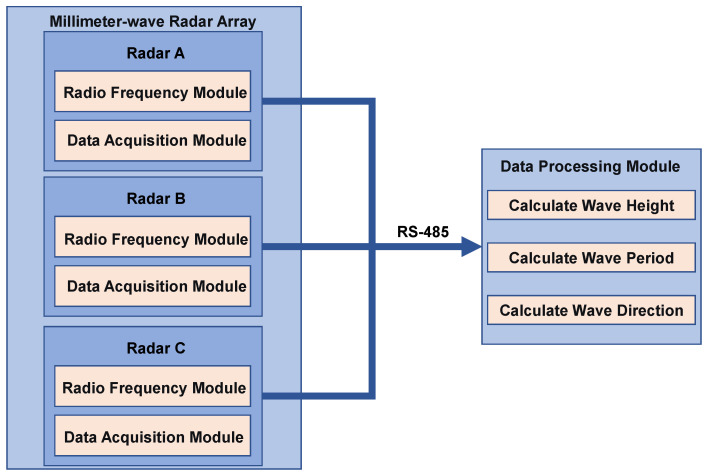
Block diagram of the 1V2I-MMWRA design.

As shown in Figure 4, the signals output by the RF module are first transmitted to the data acquisition module via the data transmission module. Within the acquisition module, range processing is performed, and the processed data are subsequently forwarded to the data processing module. To construct the array, three millimeter-wave radar units are combined in a specific “one-vertical-two-inclined” configuration to form the 1V2I-MMWRA.

#### 2.2.1. RF Module Design

The RF module of the millimeter-wave radar is designed based on the TI RF chip, constituting a compact 60 GHz millimeter-wave radar system. The core board of the RF module features dimensions of only 35×35 mm and integrates key components, including a power management integrated circuit, flash memory, and a crystal oscillator. Essential interfaces, such as JTAG (XDS110), UART, CAN-FD, SPI, SYNC, and power supply lines (3.3 V, 1.2 V, and 1.8 V), are accessible via a 1.27-mm dual-row pin header. The system input voltage is 5 V. The core board of the radar RF module integrates a 3-transmit and 4-receive microstrip antenna array, with each antenna element configured in a 1×3 subarray structure. The antenna exhibits an S11 bandwidth of 1.4 GHz at −10 dB (61.3 to 62.7 GHz) and a −6 dB bandwidth of 2.5 GHz (60.8 to 63.3 GHz). In conjunction with the employed measurement algorithms, the antenna array achieves a field of view of $\pm 60^{\circ }$ in azimuth and $\pm 25^{\circ }$ in elevation. High-precision range measurements with millimeter-level accuracy are achieved by utilizing wideband frequency modulated continuous wave signals and a high signal-to-noise ratio receiver chain.

#### 2.2.2. Design of the Data Acquisition Module

**Figure 5:**
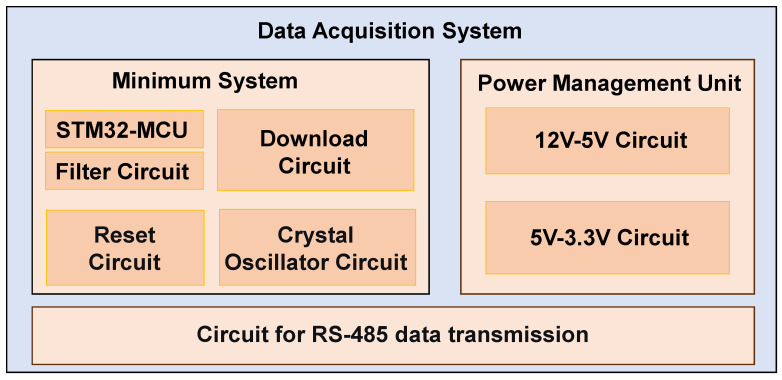
Block diagram of the data acquisition module.

As shown in Figure 5, the overall architecture of the data acquisition module. The module primarily comprises a microcontroller minimal system circuit, a power management circuit, and an RS485 data communication circuit. The detailed design configuration is described as follows:

1. Microcontroller Minimal System Design and Chip Selection
  In this study, an STM32 series microcontroller was selected as the microcontroller unit for data acquisition. The minimal system circuit comprises an external crystal oscillator circuit, a filtering circuit, a reset circuit, and a programming interface circuit. These components ensure that the microcontroller operates with a stable clock source, a high-quality power supply, and convenient programming capabilities.
2. Power Management Module Design
  The power management module primarily implements multi-stage voltage regulation and power conversion. Initially, a DC-DC non-synchronous buck converter is employed to step down the 12 V supply to 5 V, delivering a maximum output current of 3 A to meet the system’s power requirements. Subsequently, a low-dropout regulator is utilized to further regulate the 5 V rail down to 3.3 V. This stage provides a maximum output current of 800 mA, ensuring a stable power supply for the microcontroller and other low-voltage peripherals.
3. Data Transmission Module Design
  The data acquisition module is interfaced with the data processing module through an RS-485 communication link. This section utilizes a half-duplex RS-485 transceiver to ensure robust and interference-resistant data transmission, leveraging the advantages of differential signaling to mitigate electromagnetic noise. This communication scheme is characterized by its hardware simplicity and high operational reliability, making it particularly well-suited for the complex and demanding environments of offshore platforms.

Based on the aforementioned design, the data acquisition module of the millimeter-wave radar facilitates reliable and efficient signal reception, processing, and transmission. This architecture establishes a dependable foundation for the subsequent analysis and retrieval of wave characteristic parameters.

### 2.3. Principle of Millimeter-Wave Radar Ranging

Millimeter-wave radar is a sensing technology that leverages electromagnetic waves with millimeter-scale wavelengths for target detection. When the waves emitted by the radar system encounter an object, they are partially reflected back to the receiver. By acquiring and processing these reflected signals, the system can accurately determine the target’s range, velocity, and azimuth [22].

**Figure 6:**
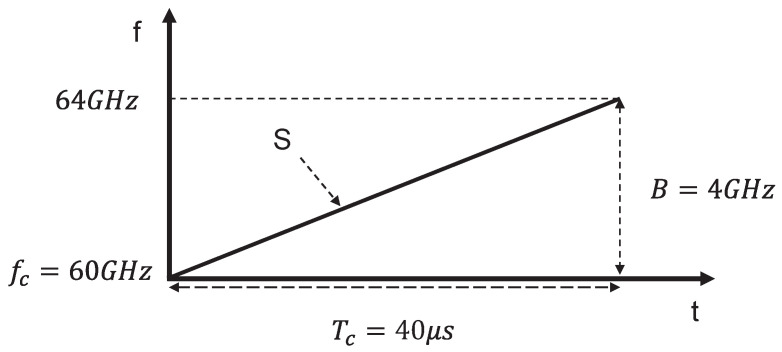
Frequency modulated continuous wave signal versus time.

In the ranging process of millimeter-wave radar, it is first necessary to generate a linear frequency modulation signal; since its frequency increases linearly with time, it is also called a Linear Frequency Modulated Continuous Wave (LFMCW) signal [23]. As shown in Figure 6, to more intuitively characterize its time-frequency properties, the frequency of the LFMCW signal can be expressed as a function of time. The signal is defined by a starting frequency $f_{c}$, a bandwidth *B*, and a chirp duration $T_{c}$. The frequency modulation slope *S*, which represents the rate of change of frequency over time, serves as a critical parameter for range determination in millimeter-wave radar systems [24].

**Figure 7:**
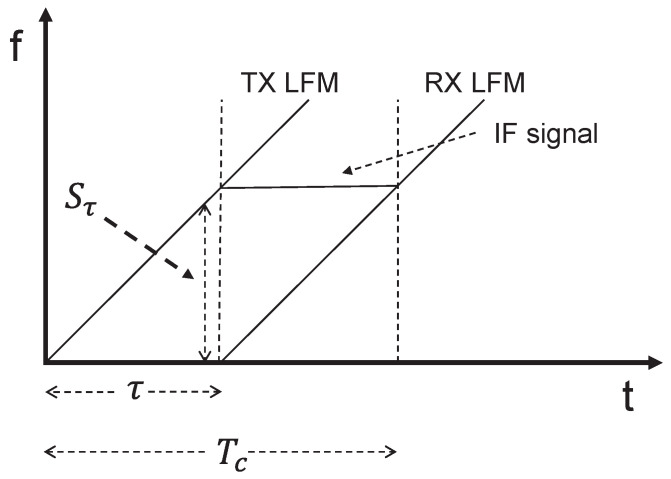
Signal mixing schematic.

After generating the LFMCW, the signal enters transmission and mixing, where the signal mixing process is shown in Figure 7, the mixer combines the transmitting antenna and receiving antenna signals to generate a new Intermediate Frequency (IF) signal. Because the receiving antenna signal undergoes a round-trip propagation, it exhibits a time delay, denoted as τ. By the time the echo returns to the radar, the frequency of the transmitting antenna signal has increased, resulting in a constant frequency difference Δf between the two signals [25]. This frequency difference maintains a direct mathematical relationship with the target range, forming the operational basis for millimeter-wave radar ranging.

The IF signal obtained through the above mixing process provides the necessary parameters for further calculation of the radar’s detection range. If the target is at a distance *r* and the millimeter-wave propagation speed is assumed to be the speed of light *c*, the time delay τ can be expressed as:

(1) $$\tau =\frac{2r}{c}$$

Based on Equation (1), the range calculation formula can be directly derived as follows:

(2) $$r=\frac{c\tau }{2}$$

Based on the characteristics of the LFMCW signal as shown in Figure 6, the frequency modulation slope *S* is defined as the rate of change of the bandwidth *B* over the modulation period $T_{c}$:

(3) $$S=\frac{B}{T_{c}}$$

Here, *S* represents the rate of change of frequency over time, that is, the rate at which the radar frequency increases from 60 GHz to 64 GHz over the period $T_{c}$. From this, the relationship between the frequency difference Δf and the time delay τ can be derived as follows:

(4) $$S=\frac{\Delta f}{\tau }$$

By substituting Equation (1) into Equation (4), the relationship between the target range *r*, the frequency difference Δf, and the modulation slope *S* can be obtained.

(5) $$r=\frac{\Delta fc}{2S}$$

Equation (5) shows that the radar’s maximum detection range is determined by the maximum detectable beat frequency $\Delta f_{max}$. Moreover, $\Delta f_{max}$ is limited by the system bandwidth *B* and the sampling frequency $F_{s}$ of the IF signal.

According to the sampling theorem, the frequency difference should satisfy $\Delta f\leq \frac{F_{s}}{2}$. If the LFMCW signal has *N* sampling points within one period, then:

(6) $$r<\frac{F_{s}c}{4S}=\frac{Nc}{4T_{c}S}=\frac{Nc}{2B}$$

Equation (6) demonstrates that the maximum detection range of the radar is governed by system parameters, including the sampling frequency, bandwidth, and modulation period. Consequently, these factors influence the determination of the radar’s installation height and its effective coverage area.

### 2.4. Millimeter-Wave Radar Array Design

**Figure 8:**
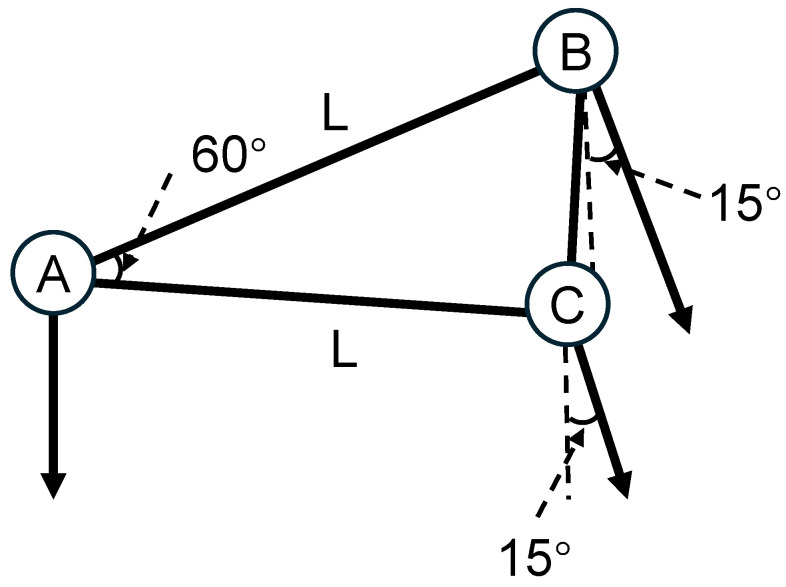
Spatial configuration of the 1V2I-MMWRA.

A 1V2I-MMWRA system comprising three millimeter-wave radar units is employed. The spatial configuration of the array as shown in Figure 8. Radar A is positioned at the center in a vertical orientation, while radars B and C are deployed on opposite sides of radar A. Collectively, the three radars form an equilateral triangular arrangement with a side length L=35 cm and an included angle of $60^{\circ }$.

To facilitate the accurate reconstruction of the three-dimensional wave surface, radars B and C are installed with a tilt along the A–B and A–C axes, respectively. Their boresight axes are inclined at an angle of $15^{\circ }$ relative to the vertical. This configuration enables the sensing beams of the three radars to achieve effective spatial complementarity and coverage overlap. Specifically, the vertically mounted Radar A primarily captures vertical fluctuations in sea surface elevation, whereas the two inclined radars, B and C, effectively acquire wave characteristics from distinct lateral directions.

The sea surface elevation data synchronously acquired by this array are fused and processed to invert wave power and directional spectra with high spatiotemporal resolution. This process enables the precise calculation of key wave parameters, including wave height, wave period, and wave direction. Consequently, this configuration effectively mitigates the inherent limitations of single vertical radar, specifically regarding directional ambiguity and the measurement of steep slopes, thereby significantly enhancing the overall accuracy of wave parameter estimation.

### 2.5. Calculation of Wave Height and Wave Period

#### 2.5.1. Zero-up-Crossing Method

**Figure 9:**
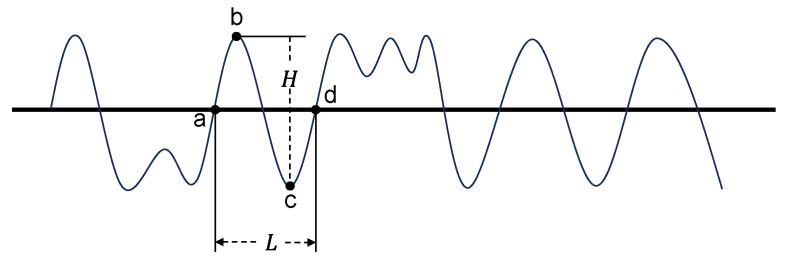
Zero-crossing method schematic.

The zero-crossing method is a widely adopted time-domain approach for estimating wave characteristic parameters. As shown in Figure 9, the ranging data acquired by the millimeter-wave radar are processed using a moving average filter to eliminate tidal variations, thereby obtaining the vertical fluctuations of the sea surface elevation. The arithmetic mean of the entire wave series serves as the mean water level.

The mean water level intersects the wave profile at multiple points. Point a, where the ascending slope of the wave crosses the mean water level, is defined as the up-crossing point. The segment of the wave profile between two successive up-crossing points, a and d, constitutes a single wave cycle, and the corresponding time interval is defined as the wave period. Furthermore, the vertical distance between the wave crest b and the wave trough c within this cycle is defined as the wave height [26].

To derive statistical wave parameters, all calculated individual wave heights are sorted in descending order. The largest value is defined as the maximum wave height, and its associated period is referred to as the maximum wave period. The arithmetic mean of the highest one-third of the wave heights is defined as the significant wave height, while the average of the entire dataset constitutes the mean wave height. Correspondingly, the mean of the wave periods associated with the highest one-third of the waves is defined as the significant wave period, whereas the average of all recorded periods is termed the mean wave period [27].

#### 2.5.2. Wave Height and Wave Period Estimation Using the Spectral Method

The fundamental principle of the spectral method involves transforming the signal from the time domain to the frequency domain to elucidate its frequency components and energy distribution. In this study, the 1V2I-MMWRA, deployed on an offshore meteorological observation tower, continuously acquires the vertical displacement time series of the sea surface via non-contact measurement. This time series captures the dynamic temporal fluctuations of the waves. By applying a Fourier transform to the displacement data, a power spectrum is derived, representing the distribution of wave energy as a function of frequency. Based on the spectral moments and frequency-domain characteristics of this spectrum, key wave parameters—such as wave height and wave period—are retrieved, thereby effectively characterizing the sea state conditions. Following the transformation of the wave surface elevation time series into the frequency domain using the Fourier transform, spectral moments are utilized to calculate wave characteristic parameters. The *n*-th order spectral moment $m_{n}$ is defined as [28]:

(7) $$m_{n}=\int_{0}^{\infty }f^{n}S\left(f\right)df,\;n=0,1,2,\ldots$$

where S(f) denotes the power spectrum, and *f* represents the frequency [29,30,31,32].

Significant wave height: $H_{s}=4\sqrt{m_{0}}$.
Mean wave height: $H_{m}=\sqrt{2\pi m_{0}}$.
Mean wave period: $T_{m}=\frac{m_{0}}{m_{1}}$.
Significant wave period: $T_{s}=1.1T_{m}$.

### 2.6. Calculation of Wave Direction

Among the established methods for estimating the wave directional spectrum, parametric estimation is widely regarded as a practical and efficient approach, with the truncated Fourier series expansion being the most straightforward variant. The fundamental principle involves utilizing the three displacement time series acquired by the 1V2I-MMWRA to derive the necessary directional parameters. Specifically, by applying the truncated Fourier series method proposed by Longuet-Higgins, the wave directional spectrum can be expressed as:

(8) D(θ,f)=S(f)·G(θ,f)

where θ represents the wave direction, D(θ,f) denotes the wave directional spectrum, S(f) represents the power spectrum, *f* is the frequency, and G(θ,f) is the directional distribution function, which is generally expressed as:

(9) $$G\left(\theta ,f\right)=\frac{1}{\pi }\left\{\frac{1}{2}+a_{1}\cos\theta +b_{1}\sin\theta +a_{2}\cos2\theta +b_{2}\sin2\theta +\ldots \right\}$$

where $a_{1}$, $a_{2}$, $b_{1}$ and $b_{2}$ are coefficients.

Equation (8) demonstrates that the estimation of the wave directional spectrum requires combining the power spectrum with the directional distribution function. To achieve this, the displacement time series corresponding to the north, west, and vertical components are processed, and their respective Fourier coefficients are computed using the Fourier transform. Since each Fourier series consists of complex coefficients (comprising both real and imaginary parts), the six Fourier components per frequency *f* are obtained: $\alpha_{nf}$, $\beta_{nf}$, $\alpha_{wf}$, $\beta_{wf}$, $\alpha_{vf}$, and $\beta_{vf}$, or in vector notation:

(10) $$\begin{array}{c} A_{nf}=\alpha_{nf}+i\beta_{nf}\\ A_{wf}=\alpha_{wf}+i\beta_{wf}\\ A_{vf}=\alpha_{vf}+i\beta_{vf} \end{array}$$

Consequently, the corresponding co-spectra and quad-spectra can be constructed, with their expressions given by:

The co-spectrum is given by: $C_{nw}=\bar{A_{nf}}\cdot \bar{A_{wf}}=\alpha_{nf}\alpha_{wf}+\beta_{nf}\beta_{wf}$.

The quad-spectrum is given by: $Q_{vn}=\bar{A_{vf}}\times \bar{A_{nf}}=\alpha_{vf}\beta_{nf}-\beta_{vf}\alpha_{nf}$.

Through the aforementioned processing, the co-spectra and quad-spectra are respectively obtained. Based on the analysis of the physical wave state, the quad-spectrum is generally taken as: $Q_{nn}=Q_{ww}=Q_{vv}=Q_{wn}=Q_{nw}=0$.

The resulting components are [33]:

(11) $$\left(\begin{array}{ccc} C_{ww} & C_{wn} & C_{wv}\\ C_{nw} & C_{nn} & C_{nv}\\ C_{vw} & C_{vn} & C_{vv} \end{array}\right)\;\mathrm{and}\;\left(\begin{array}{ccc} 0 & 0 & Q_{wv}\\ 0 & 0 & Q_{nv}\\ Q_{vw} & Q_{vn} & 0 \end{array}\right)$$

Therefore, combining the given components yields [34]:

(12) $$\begin{array}{c} a_{1}=\frac{Q_{nv}}{\sqrt{\left(C_{nn}+C_{ww}\right)C_{vv}}}\\ b_{1}=\frac{-Q_{wv}}{\sqrt{\left(C_{nn}+C_{ww}\right)C_{vv}}}\\ a_{2}=\frac{C_{nn}-C_{ww}}{C_{nn}+C_{ww}}\\ b_{2}=\frac{-2C_{nw}}{C_{nn}+C_{ww}} \end{array}$$

Consequently, the directional distribution function G(θ,f) can be calculated. Therefore, by combining the known power spectrum S(f) with the directional distribution function G(θ,f), the wave directional spectrum is derived. The wave direction corresponding to the maximum PSD of the directional spectrum is identified as the observed wave direction.

## 3. Results and Discussion

### 3.1. Determination of Wave Characteristic Parameters Using the Zero-Crossing Method

**Figure 10:**
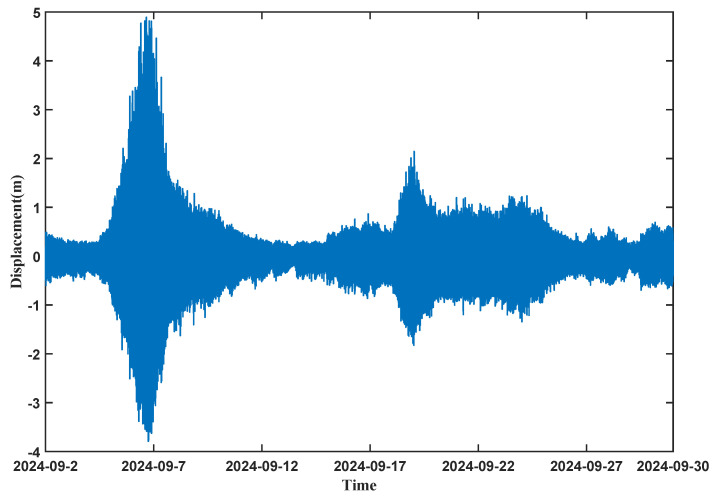
Wave surface displacement from 2 to 30 September 2024.

As shown in Figure 10, sea surface elevation data were collected using the 1V2I-MMWRA from 2 to 30 September 2024. In order to guarantee the representativeness and timeliness of the wave statistics, this study divided the continuous observation data into 10-min segments for analysis. The zero-crossing method was then employed to calculate the wave height and wave period for each 10-min segment.

**Figure 11:**
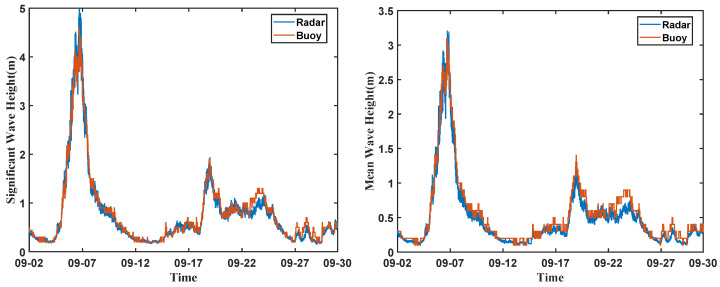
Significant wave height and mean wave height using the zero-crossing method.

Figure 11 presents the significant and mean wave heights derived using the zero-crossing method, in the figure, “Radar” and “Buoy” represent the data measured by 1V2I-MMWRA and air–sea interface flux buoy, respectively. It can be observed that the results obtained from the 1V2I-MMWRA are highly consistent with the in situ buoy measurements in terms of the overall trend. Furthermore, the two datasets exhibit a strong correlation in amplitude variations. Specifically, the RMSE between the 1V2I-MMWRA and buoy measurements for the significant wave height and mean wave height were 0.13 m and 0.11 m, respectively. The Mean Absolute Error (MAE) were 0.08 m for both parameters. The RMSE values for the significant and mean wave heights measured by the buoy and the 1V2I-MMWRA are relatively small. This demonstrates that the 1V2I-MMWRA possesses the capability to accurately retrieve sea surface wave parameters. It is particularly noteworthy that during the typhoon influence from 6 to 8 September 2024, when wave conditions fluctuated drastically, the 1V2I-MMWRA was still able to stably capture subtle variations in wave height. The obtained results remained almost synchronized with the buoy data. This result validates that the 1V2I-MMWRA exhibits high measurement precision in steady sea conditions while retaining superior reliability even under complex and dynamic circumstances.

**Figure 12:**
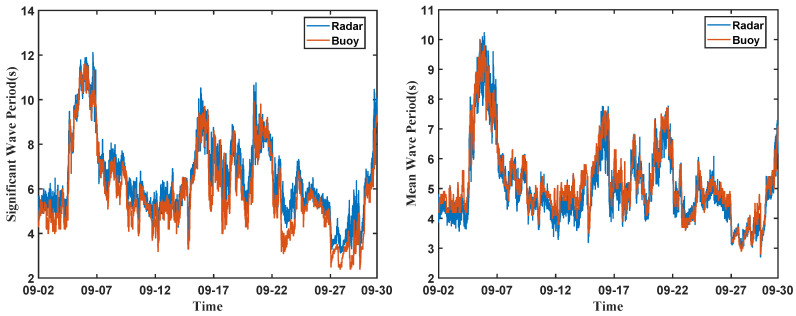
Significant wave period and mean wave period using the zero-crossing method.

Therefore, it can be concluded that the 1V2I-MMWRA, based on the zero-crossing method, is capable of performing high-precision, continuous, and real-time measurements of significant and mean wave heights. It not only accurately reflects the statistical characteristics of waves but also maintains measurement stability under extreme weather conditions. AS shown in Figure 12, the significant and mean wave periods derived using the zero-crossing method. It can be observed that the results calculated by the 1V2I-MMWRA are consistent with the buoy measurements regarding the overall trend. Furthermore, the two datasets exhibit a good correspondence in terms of temporal variations. Specifically, the RMSEs between the 1V2I-MMWRA and buoy measurements for the significant and mean wave periods were 0.81 s and 0.46 s, respectively. The MAEs were 0.6 s and 0.3 s, respectively.

This demonstrates that the 1V2I-MMWRA is capable not only of accurately acquiring wave height information but also of achieving high precision and stability in wave period extraction. In particular, during the complex sea states characterized by the typhoon passage and drastic wave fluctuations from 6 to 8 September 2024, the 1V2I-MMWRA was still able to accurately capture the variation patterns of wave periods. The calculated results remained highly consistent with the buoy observations. This demonstrates that the 1V2I-MMWRA, based on the zero-crossing method, possesses excellent dynamic response capability and environmental adaptability, enabling it to maintain the accuracy and consistency of measurement results even under complex sea states. Consequently, the combination of the 1V2I-MMWRA and the zero-crossing method is proven to be a highly accurate and real-time technique for measuring wave periods.

### 3.2. Estimation of Wave Parameters Using Spectral Estimation Method

**Figure 13:**
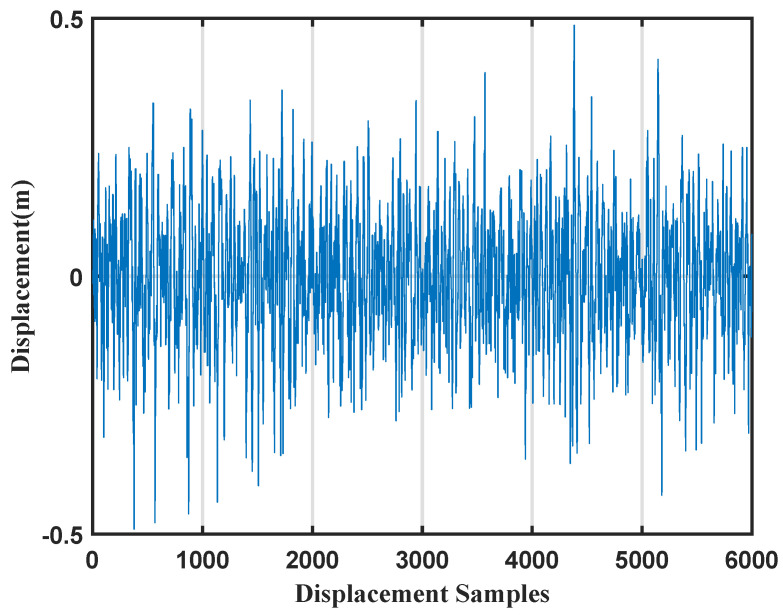
Vertical wave surface displacement over a ten-minute period.

As shown in Figure 13, the sea surface elevation time series measured by radar A during a 10-min interval at 00:00 (Beijing Time, UTC+8) on 2 September 2024. Subsequently, spectral estimation was performed on this time series using the Fast Fourier Transform.

**Figure 14:**
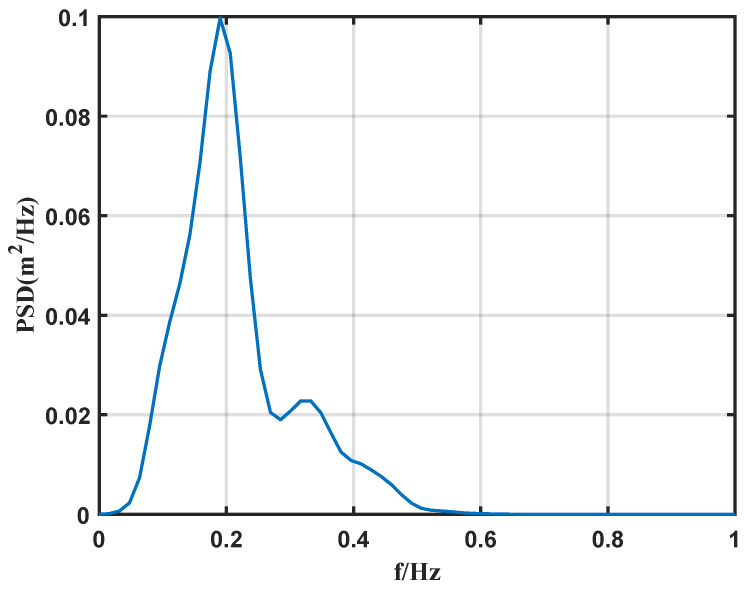
Power Spectral Density.

As shown in Figure 14, the power spectrum of the 10-min sea surface elevation time series, obtained using the spectral estimation method employed in this study.

**Figure 15:**
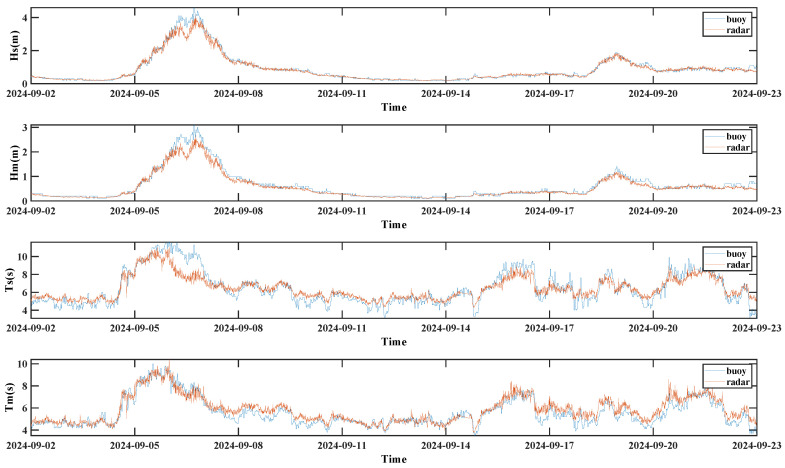
Wave characteristic parameters derived using the spectral estimation method.

As shown in Figure 15, the wave parameters derived using the spectral estimation method are displayed. The results indicate that the 1V2I-MMWRA data align well with the buoy measurements regarding the general trend. Both datasets exhibit strong agreement regarding temporal evolution and fluctuation trends, demonstrating the high accuracy and reliability of the 1V2I-MMWRA for wave parameter monitoring.

The numerical analysis yields RMSEs of 0.20 m and 0.16 m for significant and mean wave heights, and 1.07 s and 0.74 s for significant and mean wave periods. The corresponding MAEs are 0.12 m, 0.10 m, 0.79 s, and 0.55 s. However, the significant wave period exhibits lower accuracy compared to the other results. This discrepancy is mainly associated with the complex sea conditions caused by the typhoon. In particular, during the typhoon passage from 6 to 8 September, the wave energy distribution underwent significant changes under the influence of strong winds. Under typhoon conditions, wave spectrum estimation requires accounting for nonlinear wave interactions. However, since the spectral estimation method employed in this study relies on linear analysis to estimate wave characteristic parameters, the discrepancies between the parameters measured by the 1V2I-MMWRA and the buoy observations are significantly larger than those observed during non-typhoon periods.

**Figure 16:**
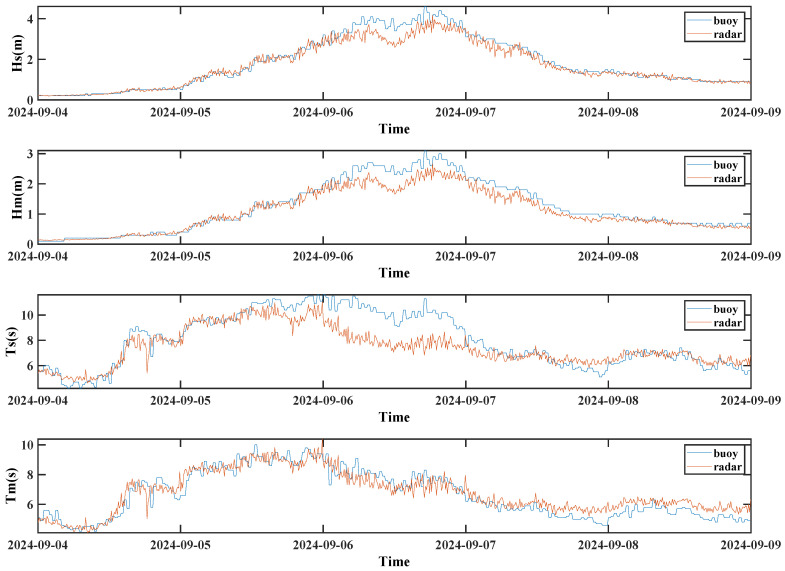
Wave characteristic parameters derived using the spectral estimation method during the typhoon period.

To further investigate this phenomenon, this study performs a separate analysis of the wave parameters obtained using the spectral estimation method during the typhoon event. As shown in Figure 16, the 1V2I-MMWRA captured the wave parameters during the typhoon event at the offshore tower. During the passage of the Level-17 Super Typhoon “Yagi” from 6 to 8 September 2024, the observation site (approx. 173 km from the typhoon center) was significantly affected. Throughout this event, the 1V2I-MMWRA achieved continuous observation of the wave parameter variations.

This study compares the wave characteristic parameters retrieved by the spectral estimation method (based on 1V2I-MMWRA observations) with in situ measurements from an air–sea interface flux buoy. The results demonstrate that the wave heights and periods retrieved by the millimeter-wave radar during the typhoon passage exhibit temporal trends that align closely with the buoy observations.

Quantitative analysis indicates that during the typhoon, the RMSEs for significant and mean wave heights were 0.31 m and 0.25 m, respectively, with MAEs of 0.20 m and 0.17 m. Although these error metrics increased compared to non-typhoon periods, the Pearson correlation coefficients for both parameters reached 0.98, demonstrating exceptional consistency.

Regarding the significant wave period, the deviation was more pronounced, with an RMSE of 1.20 s and an MAE of 0.85 s; nevertheless, the Pearson correlation coefficient remained robust at 0.87. In contrast, the estimation of the mean wave period maintained higher precision, achieving a correlation coefficient of 0.93 (surpassing that of the significant wave period), with an RMSE of 0.61 s and an MAE of 0.49 s. Collectively, these results indicate that the 1V2I-MMWRA is capable of measuring wave characteristic parameters with relative accuracy even under extreme weather conditions.

The aforementioned analysis demonstrates that the temporal variation trends of the wave characteristic parameters obtained by the 1V2I-MMWRA are highly consistent with the buoy observations, exhibiting strong agreement in their fluctuation characteristics. Regarding the wave parameters, although certain deviations occurred during the typhoon passage, a robust correlation was maintained throughout the observation period.

### 3.3. Analysis of Wave Directional Spectrum Using Parametric Method

**Figure 17:**
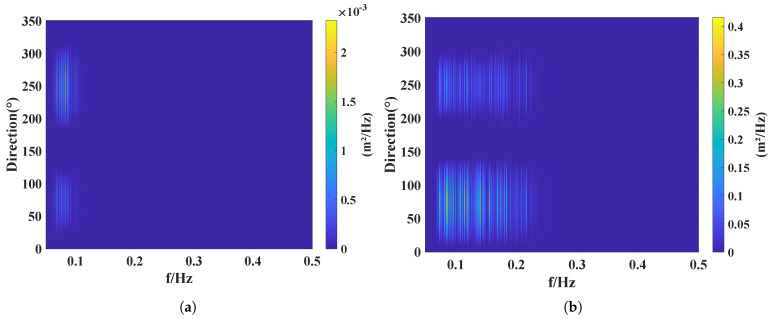
Wave directional spectrum: (**a**) on 2 September 2024; (**b**) under typhoon conditions on 6 September 2024.

In this paper, the wave directional spectrum is analyzed using the parametric estimation method. As shown in Figure 17a, the measured directional spectrum for 2 September 2024. The distribution exhibits distinct bimodal characteristics, which corroborate the two separated peaks observed in the power spectrum. This indicates that the observed sea area was influenced by the superposition of wind waves and swell during this period, resulting in a wave energy distribution characterized by a clear bimodal structure.

Based on the directional spectrum analysis, the PSD of the dominant peak exceeds 0.002 m^2^/Hz. The wave energy is primarily concentrated within the directional sector of $225^{\circ }$ to $275^{\circ }$, indicating a distinct directional concentration of the wave energy. The dominant peak power corresponds to a direction of $250^{\circ }$, indicating a dominant wave direction of $250^{\circ }$, which aligns with the on-site visual estimates. Similar to wave height and wave period, the PSD of the wave directional spectrum is also significantly affected by the typhoon. Figure 11 shows that the significant wave height was below 0.5 m on 2 September 2024, indicating relatively calm sea conditions with minor surface fluctuations. By comparing Figure 17a,b, it can be observed that Under calm sea conditions, wave frequencies were primarily concentrated below 0.1 Hz with relatively low PSD. As the typhoon approached, sea surface fluctuations gradually intensified; the dominant frequency shifted from below 0.1 Hz to approximately 0.2 Hz, and the PSD correspondingly increased from less than 0.1 to about 0.4, exhibiting a pronounced enhancement trend.

**Figure 18:**
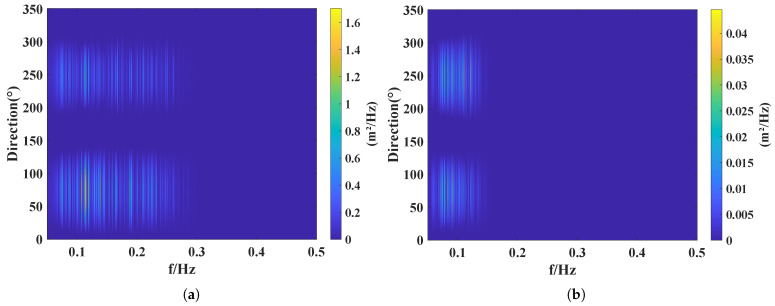
Wave directional spectrum: (**a**) during the peak intensity of the typhoon; (**b**) after the passage of the typhoon.

Figure 18a captures the moment when the sea surface fluctuations reached their maximum during the typhoon passage. As shown in the figure, the maximum PSD attained a value of 1.6 m^2^/Hz. By combining Figure 17b and Figure 18a, it can be observed that during the typhoon period, the dominant peak of the wave spectrum corresponds to directions concentrated between $50^{\circ }$ and $100^{\circ }$. The calculated principal wave direction is $90^{\circ }$, which is in good agreement with the wave direction of $105^{\circ }$ measured by the air–sea flux buoy. As shown in Figure 18b, the wave directional spectrum after the typhoon had passed. It can be seen that the wave frequency decreases and the PSD is reduced at this stage. This indicates that under the influence of the typhoon, the wave frequency continuously increased and the wave energy was correspondingly enhanced.

### 3.4. Survey and Comparison of Measurement Devices

This study conducted a technical specification comparison for conventional non-contact wave measurement devices, among which the technical specifications of the 1V2I-MMWRA were derived based on comparative analysis with an air–sea interface flux buoy, and the results are shown in Table 2. Among the three, stereo vision has the highest measurement accuracy, but stereo vision cannot achieve all-weather measurement and has high deployment costs. Although X-band marine radar measurement can achieve all-weather measurement, its power consumption is high and its measurement accuracy is lower than that of stereo vision. The 1V2I-MMWRA can achieve easy to deploy, low power, all weather measurement under the premise of guaranteeing a certain level of accuracy.

## 4. Conclusions

The millimeter-wave radar system designed in this study utilizes the TI RF chip as its core to construct the RF module. This RF module, combined with a data acquisition module, constitutes the complete radar module. The RF module is responsible for generating and receiving millimeter-wave signals. It performs the transmission of LFMCW signals, echo reception, and mixing processing, thereby outputting IF signals containing target range information. After receiving the IF signal, the data acquisition module processes it to resolve the target range. At the system level, this study proposes a one-vertical-two-inclined millimeter-wave radar array structure. This configuration enables the three radars to form spatially complementary fields of view for wave detection, thereby achieving the precise capture of wave directional information.

Upon finalizing the design of the 1V2I-MMWRA, field experiments were launched based at the tower of Bohe Marine Meteorological Field Experiment Base, CMA, located in the South China Sea. The radar system was employed to conduct systematic monitoring of wave parameters around the offshore observation tower. The primary measurement parameters include significant wave height, mean wave height, significant wave period, mean wave period, and wave direction. To validate the reliability and accuracy of the measurement results from the 1V2I-MMWRA, this study conducted a comparative analysis with the observational data acquired from an air–sea flux buoy deployed near the meteorological observation tower.

Experimental results indicate that regardless of whether the zero-crossing method or the spectral estimation method is applied, the wave characteristic parameters obtained by the 1V2I-MMWRA maintain good consistency with the buoy measurements in terms of overall trends. This demonstrates that the radar system possesses high observational reliability and stability. Specifically, the results for significant wave height, mean wave height, significant wave period, and mean wave period obtained using the zero-crossing method exhibited higher agreement with the buoy data. The RMSEs were 0.13 m, 0.11 m, 0.81 s, and 0.46 s, respectively. The deviation for wave height was merely at the centimeter level, while the deviation for wave period was within the sub-second range. This fully demonstrates that the 1V2I-MMWRA has achieved a high level of precision in measuring wave heights and periods.

In contrast, the wave characteristic parameters calculated using the spectral estimation method exhibited lower consistency with the buoy data. The corresponding RMSEs were 0.20 m, 0.16 m, 1.07 s, and 0.74 s. Although the errors increased in numerical terms, the deviation for wave height remained at the centimeter level, and the deviation for the mean wave period stayed within a reasonable millisecond-scale range. Compared to the zero-crossing method, the precision of the spectral estimation method was slightly lower, with RMSE values approximately 0.07 m, 0.05 m, 0.26 s, and 0.28 s higher. One possible reason for this discrepancy is the interference effect of the typhoon weather. During the typhoon passage, strong winds and complex wave fields cause significant changes in wave spectral characteristics, thereby affecting the computational stability of the spectral estimation method.

In the typhoon period, calculations using the spectral estimation method yielded RMSEs of 0.31 m, 0.25 m, 1.20 s, and 0.61 s for significant wave height, mean wave height, significant wave period, and mean wave period. While wave height deviations stayed at the centimeter level and the mean wave period deviation was in the sub-second range, the significant wave period estimation was less accurate than the other parameters. Nevertheless, the overall errors fell within an acceptable physical range.

The analysis of the wave directional spectrum reveals that during periods of calm sea, wave frequency and PSD both exhibit low values. Under the influence of the typhoon, both wave frequency and PSD increased significantly, with the maximum PSD reaching 1.6 m^2^/Hz during the observation experiment. In addition, the wave directions observed in this study were generally consistent with those measured by the air–sea flux buoy, albeit with certain discrepancies in specific numerical values.

The results of this study demonstrate the feasibility of the 1V2I-MMWRA for measuring wave characteristic parameters, providing important technical support for ocean forecasting, ocean engineering, and maritime safety. It was found that when the spectral estimation method is used, its estimation accuracy is affected under non-stationary conditions. Although the wave directional distribution remains generally consistent in the inversion results, issues regarding insufficient numerical accuracy still persist. In the future, parameter calibration will be conducted in laboratory wave tanks. By designing and adjusting different array geometric configurations, we will analyze the limitations of wave direction estimation caused by array geometry. We will collect more experimental data under high-wind conditions to analyze the limitations of wave direction estimation caused by water surface scattering. We will also collect more wave observation data under low sea states to specifically analyze the measurement accuracy of the method presented in this study when wave patterns are indistinct or weak. Furthermore, we will further optimize the wave direction estimation method and improve the accuracy of wave direction estimation. Finally, validation experiments will be carried out in more sea areas using fixed and shipborne underway platforms to verify performance across different sea areas, platforms, and sea states.

## Figures and Tables

**Table 1:** Key parameters of the single millimeter-wave radar module.

Parameter	Specification
Sampling frequency	10 Hz
Operating frequency	60 GHz
Power Consumption	2.83 W
Operating Voltage	12 V
Range Accuracy	0.001 m
Measurement Range	0 to 60 m

**Table 2:** Comparison of Technical Specifications.

	Accuracy of Wave Height (m)	Accuracy of Wave Period (s)	Power Consumption	All-Weather Capability	Deployment Cost
1V2I-MMWRA	⩽0.2	⩽0.9	Low	Yes	Low
Stereo Vision	⩽0.1	⩽0.5	Medium	No	High
X-Band Marine Radar	⩽0.6	⩽0.7	High	Yes	High

## Data Availability

The data presented in this study are available on request from the corresponding author.

## Abbreviations

The following abbreviations are used in this manuscript:

1V2I-MMWRA	One-Vertical-Two-Inclined Millimeter-Wave Radar Array
RMSE	Root-Mean-Square Error
MLM	Maximum Likelihood Method
EEV	Extended Eigenvector Method
BDM	Bayesian Directional Spectrum Estimation Method
RF	Radio Frequency
LFMCW	Linear Frequency Modulated Continuous Wave
IF	Intermediate Frequency
PSD	Power Spectral Density
MAE	Mean Absolute Error
